# A Multidisciplinary Approach to the Management of Eales Disease: A Case Report and Review of the Literature

**DOI:** 10.3390/jpm14030235

**Published:** 2024-02-22

**Authors:** Maria Filoftea Mercuț, Oana Maria Ică, Cornelia Andreea Tănasie, Răzvan Mercuț, Carmen Luminița Mocanu, Andreea Mihaela Nicolcescu, Ciprian Danielescu

**Affiliations:** 1 Department of Ophthalmology, University of Medicine and Pharmacy of Craiova, 200349 Craiova, Romania; maria.mercut@umfcv.ro (M.F.M.); carmen.mocanu@umfcv.ro (C.L.M.); 2 Department of Dermatology, University of Medicine and Pharmacy of Craiova, 200349 Craiova, Romania; 3 Department of Physiology, University of Medicine and Pharmacy of Craiova, 200349 Craiova, Romania; 4 Department of Plastic Surgery, University of Medicine and Pharmacy of Craiova, 200349 Craiova, Romania; razvan.mercut@umfcv.ro; 5 Department of Ophthalmology, Clinical County Emergency Hospital, 200642 Craiova, Romania; nicolcescua@gmail.com; 6 Department of Ophthalmology, University of Medicine and Pharmacy Grigorie T. Popa Iasi, 700115 Iasi, Romania; ciprian.danielescu@umfiasi.ro

**Keywords:** Eales disease, *Mycobacterium tuberculosis*, epimacular membrane, retinal vasculitis

## Abstract

Eales disease manifests as an obliterative periphlebitis affecting the retina; it originates from the periphery and progresses posteriorly. It is characterized by retinal vessel wall inflammation, ischemia, and retinal neovascularization. In this report, we present the case of a 34-year-old male who attended our clinic with a sudden blurring of vision in his right eye. A diagnosis of bilateral retinal vasculitis with vitreal hemorrhage was ascertained in his RE. A dilated ocular fundus examination revealed perivenous sheathing of the peripheral vessels in both eyes. Fluorescein angiography indicated dye staining, vessel obliteration, capillary drop-out, areas of non-perfusion and the formation of new vessels. Laboratory tests revealed positive results for Borrelia; a PPD skin test and QuantiFERON TB assay were also positive. The patient underwent bilateral retinal laser pan-photocoagulation, followed by systemic treatment with oral steroids, cephazoline, isoniazid, azathioprine, and entecavir. The steroid dose was progressively reduced over 10 months; the treatment with azathioprine continues, as we are monitoring the patient over the long term. After 3 months, the vasculitis had regressed without any vitreal hemorrhage recurrence. Vision acuity improved from 0.4 to 1 in the patient’s right eye. A multidisciplinary approach, which included collaborative management with gastroenterology, infectious disease, pulmonology, and rheumatology specialists, was essential for the diagnosis, treatment, and long-term follow up of the patient.

## 1. Introduction

Eales disease is an idiopathic inflammatory venous occlusive disease. It is usually bilateral, primarily affecting the peripheral retina of young male individuals. The hallmark signs of Eales disease include perivascular phlebitis, peripheral non-perfusion, and neovascularization. Clinical manifestations include vitreous hemorrhage; macular edema; epiretinal membrane; subretinal fibrosis; and tractional retinal detachment due to the occlusive nature of the disease, leading to extensive areas of neovascularization [1].

Inflammation is a key factor in the pathogenesis of the disease as it leads to the disruption of the blood–retina barrier, increasing its permeability. This results in the passage of circulating inflammatory cells into the retinal layers. In response, reactive oxygen species (ROS) and reactive nitrogen species (RNS) are generated at the retinal level. Many studies have revealed that ROS and RNS are important mediators of inflammation and ischemia, emphasizing the role of oxidative stress in promoting retinal vasculitis [2].

The role of angiogenesis in Eales disease is not yet fully understood. A study conducted by Murugeswari et al. reported increased vitreous levels of IL-6, IL-8, MCP-1, and vascular endothelial growth factor (VEGF), suggesting that they could act as key regulators of neovascularization in Eales disease [3]. The prolonged expression and release of VEGF at the retinal level has a role in the breakdown of the blood–retina barrier and the proliferation of retinal neovessels [4]. New vessels arise at the junction between the non-perfused and perfused retina, leading to recurrent hemorrhages in the vitreous body.

The etiology of the disease remains unclear, possibly due to its multifactorial origin. Various clinical studies have linked the disease to a history of exposure to tuberculosis and hypersensitivity to tubercular protein. The presence of Mycobacterium TB has been detected in epiretinal membranes obtained from patients with Eales disease [5,6]. Madhavan et al. analysed tissues of ERM from twenty-three patients with Eales disease and twenty-seven non-Eales disease patients as controls, using the nPCR technique. They reported that eleven (47.8%) epiretinal membranes from twenty-three patients with Eales disease tested positive for the M. tuberculosis genome. The difference between the Eales disease group and the control groups was statistically significant (*p* = 0.001), indicating an association between M. Tuberculosis and Eales disease [5]. A recent meta-analysis that included forty-seven studies involving 3557 patients reported that approximately 83% of the patients exhibited a positive Mantoux reaction; the tuberculosis genome was detected in 31% of patients with Eales disease using a polymerase chain reaction [7].

The management of Eales disease is dictated by the stage. The treatment options include observation, medical therapy, laser photocoagulation and pars plana vitrectomy. Systemic corticosteroids are the primary treatment option at the inflammatory stage to control retinal vasculitis [1]. Intravitreal triamcinolone may be used as an adjuvant in the presence of macular edema. Immunosuppressive drugs may be used when unacceptable side effects arise due to corticotherapy.

Laser photocoagulation is required in the proliferative stage of Eales disease. Vitreoretinal surgery is required for cases with persistent vitreous hemorrhages or tractional retinal detachment [8].

In this study, we present a complex case of a young male with bilateral Eales disease that was associated with other pathologies; these represented a challenge to diagnosis and treatment.

## 2. Clinical Case Description

### 2.1. Patient Anamnesis

A 34-year-old male was referred to our clinic complaining of decreased and blurred vision for two weeks with floaters in the right eye. The patient denied any similar ocular symptoms in the past. The patient’s medical history revealed a prior hepatitis B viral infection (for which the patient did not receive any treatment) and a tick bite, which occurred over a year prior.

### 2.2. Results of Clinical and Laboratory Examinations

At presentation (January 2023), the best corrected visual acuity was 0.4 in the patient’s right eye and 1 in his left eye, with intraocular pressure of 12 mmHg in both eyes. The examination of the anterior segment in both eyes was normal.

**Figure 1:**
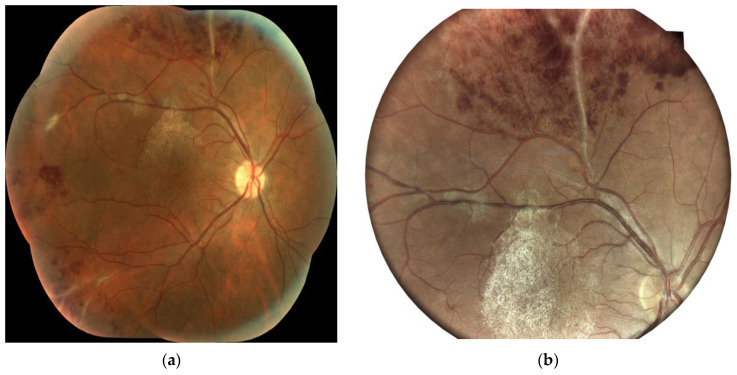
Ocular fundus in the right eye of a 34-year-old male patient: (**a**) central view and (**b**) periphery details (vitreous hematic cells; epimacular membrane; dilated veins; perivascular sheathing; peripheral focal occlusion; dotted and flame-shaped hemorrhages; and extensive non-perfused areas of the capillaries giving rise to new vessels).

**Figure 2:**
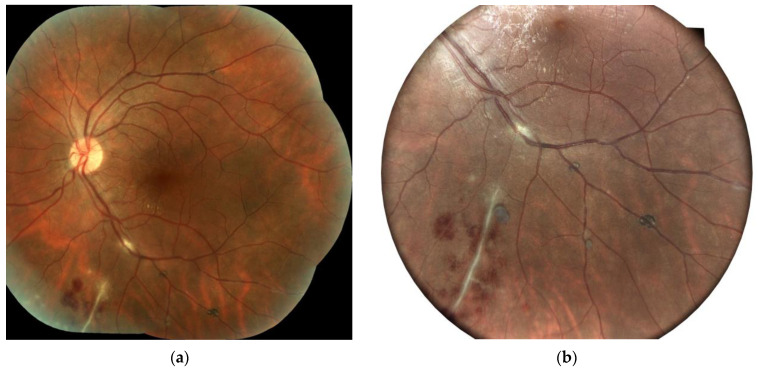
Ocular fundus in the right eye of a 34-year-old male patient: (**a**) central view and (**b**) periphery details (epimacular membrane; dilated veins; perivascular sheathing; peripheral focal occlusion; and retinal and preretinal neovascularization).

A dilated fundus examination of both eyes (Figure 1 and Figure 2) revealed a vitreous body with hematic cells+; a thick epimacular membrane; dilated veins with perivascular sheathing and focal occlusions in the periphery; venous beading; dotted and flame-shaped intraretinal hemorrhages; and prominent retinal and preretinal neovascularization in the vitreous body with a sea-fan aspect.

**Figure 3:**
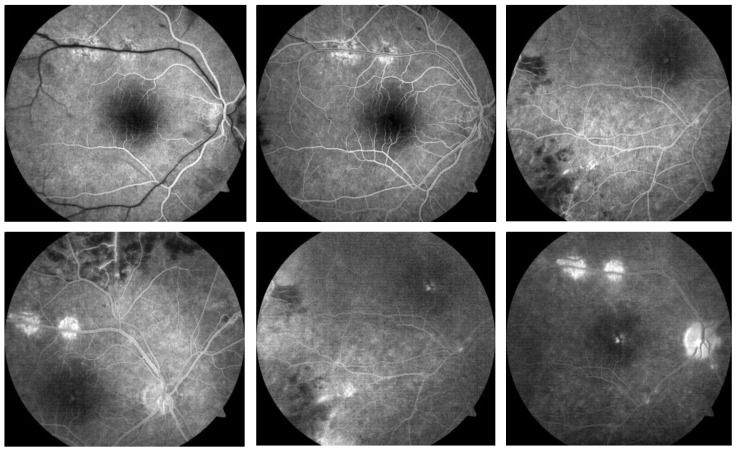
Fluorescein angiography of the right eye of a 34-year-old male with Eales disease. There were well-delineated areas of non-perfusion in the arterial phase. The early venous phase exhibited staining of the inflamed vessels, hyperfluorescence of the optic disc, and newly formed vessels. We observed leakage at the level of the new vessels and the macula, as well as staining of the vessels during the late phase.

Fundus fluorescein angiography was performed for both eyes. The arterial phase revealed sharply demarcated areas of non-perfusion. The early venous phase revealed staining of inflamed veins, well-delineated areas of non-perfusion, and hyperfluorescence of the disc in the area of the newly formed retinal vessels. The late phase demonstrated leakage of the dye from new vessels, significant staining of the vessels, and macular leakage (Figure 3).

**Figure 4:**
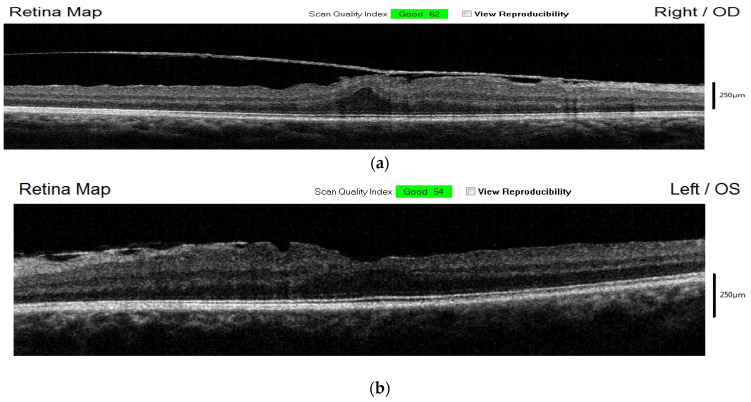
(**a**) OCT of right eye, revealing thick epimacular membrane with traction; (**b**) OCT of left eye revealing epimacular membrane.

OCT of the macula revealed a thick epiretinal membrane in both eyes. This was associated with mild retinal traction in the right eye and a bilateral posterior vitreous detachment (Figure 4).

The patient was admitted into hospital after the ophthalmological evaluation for further diagnoses. The patient had not presented with any accompanying symptoms prior to the current episode.

A comprehensive systemic workup was performed. The standard systemic examinations (ALAT, ASAT, CBC, glucose, protein electrophoresis, ESR and CRP) were within normal limits. The initial investigation also included serum angiotensin-converting enzyme (ACE) and serum lysozyme examinations; these were within a normal range.

We performed a complete panel of examinations for various infectious diseases (Table 1), which revealed positive results for herpes simplex virus (HSV1) IgG, cytomegalovirus (CMV) IgG, and Borrelia IgG and IgM. Due to the positive HSV1 IgG and CMV IgG results, we tested HSV1 DNA and CMV DNA; both were negative.

The patient underwent a pneumological examination to establish an etiology. This included a chest X-ray, which was normal, and a Mantoux test, which was positive (17 mm). A QuantiFERON TB Gold test was also positive, with a value of 3.87 UI/mL (normal range < 0.35). A ganglionar ultrasound revealed axillary and inguinal adenopathies with a reactive aspect. Magnetic resonance imaging (MRI) of the brain and orbit with contrast was normal.

Taking into account all the clinical and angiographic assessments, an early diagnosis of bilateral retinal vasculitis was ascertained. We subsequently initiated laser retinal photocoagulation in both eyes.

Due to the complex associated pathologies of the patient and the possible side effects that could arise after introducing a systemic corticosteroid treatment, we created a multidisciplinary team to further investigate the patient. This comprised a rheumatologist, a pulmonologist, a gastroenterologist and an infectious disease doctor.

An AFB sputum test was performed during the pneumology consultation; this was negative. Ganglionary tuberculosis was suspected, so a ganglionar biopsy was performed. This revealed a non-specific infectious adenitis, which eliminated ganglionary tuberculosis. The recommended treatment if immunosuppressant treatment was considered to be necessary was the prophylactic isoniazid.

The infectious disease consultation established a diagnosis of a chronic hepatitis B virus as well as seropositivity for Borrelia burgdorferi, as the patient did not meet the diagnostic criteria for Lyme disease. The recommended treatment was ceftriaxone 1 g/12 h for 14 days.

The gastroenterologist performed a FibroScan test, which revealed a F0–F1 fibrosis index. The diagnosis of a chronic hepatitis B virus was established with the recommendation of entecavir treatment, if immunosuppressant treatment was initiated.

During the rheumatology consultation, comprehensive serologic testing for autoimmune diseases was performed (C3 = 117.80 mg/dL; C4 = 25.67 mg/dL; lupic cells = negative; rheumatoid factor < 8 U/mL; autoimmune disease profile blot 18 = negative, pANCA = negative). The recommended treatment was therapy with azathioprine.

At this stage, a diagnosis of bilateral proliferative Eales disease was established based on the clinical findings, laboratory results, and the results of the multidisciplinary exams.

**Figure 5:**
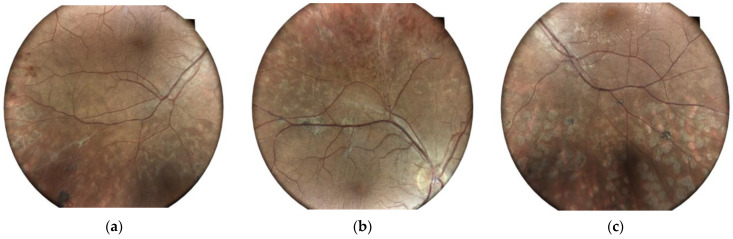
Right eye (**a**,**b**), and left eye (**c**), revealing resolved vasculitis and new-vessel regression after laser photocoagulation.

The local treatment consisted of laser scatter retinal photocoagulation in both eyes (Figure 5).

The multidisciplinary approach enabled us to ascertain an individualized care plan. The patient received intravenous treatment with methylprednisolone (1 g/day for 3 days) followed by oral corticosteroids, with a slow reduction in the doses. The initial oral prednisolone dose was 1 mg/kg/day; this was reduced by 5 mg every week until a dose of 50 mg was reached. After a week of 50 mg per day, the dose was reduced by 5 mg every 2 weeks until a dose of 20 mg was reached. After a week of 20 mg/day, the dose was reduced by 2.5 mg every week until a dose of 10 mg was reached. After 2 weeks of 10 mg/day, the dose was reduced by 1 mg every week until a dose of 5 mg/day was reached. After 5 mg/day for 4 weeks, a dose of 5 mg every 2 days was recommended for another 4 weeks. Lastly, the patient received 5 mg every 3 days for a month. We initiated immunosuppressive therapy with azathioprine, 50 mg twice daily, according to the rheumatologist’s recommendation. The patient also received prophylactic anti-tubercular therapy with isoniazid due to the positive Mantoux and QuantiFERON TB Gold tests, as well as the presence of polyadenopathies. Due to the risk of hepatitis B virus reactivation during corticosteroid therapy, entecavir (0.5 mg once daily) was initiated as per the gastroenterologist’s recommendation.

Progress was favorable after the initiation of the medical treatment alongside the laser treatment. After three months, the vasculitis regressed and the patient was clinically stable. The visual acuity was 20/20 and there was no recurrence of vitreous hemorrhages. The patient received oral steroids for 10 months and continues the treatment with azathioprine as we are monitoring the patient over the long term. We continue to review the patient every two months.

## 3. Discussion

Several studies have focused on the etiopathology of and clinical aspects encountered in Eales disease, as well as the management and treatment of the disease. We searched the PubMed database using the keyword “Eales disease” with a publication date within the last 10 years. We identified 60 articles that referred to Eales disease (reviews, case reports and original articles). Of these, we selected 20 case reports; a few are presented in Table 2.

The etiology of Eales disease remains controversial and is poorly understood. Although the disease is considered to be largely idiopathic, exposure to tuberculosis and hypersensitivity to tuberculoprotein are presumed to be related to this pathology, due to the fact that no specific cause has been clearly elucidated. There are extensive medical studies examining the association between Eales disease and tuberculosis [1,5,14].

Rajpal et al. conducted an extensive study on 65 cases of Eales disease, reporting M. tuberculosis DNA PCR positivity in 38.7% of the patients [15]. Mycobacterium species were isolated from the epiretinal membranes and vitreous humors of patients with Eales disease, revealing a statistically significant association between Eales disease and Mycobacteria [5,14].

Another retrospective study performed by Gupta et al. revealed that one or more tests for TB were positive in patients with Eales disease. This indicates a potential role of TB as an etiology for this disease [16]. Zhao et al. reported that 83% of the patients included in their analysis presented a positive Mantoux test [7]. In our study, we obtained similar results; our patient presented positive Mantoux and QuantiFERON TB Gold tests

Immunity-related mechanisms have also been included in various studies, revealing an association between HLA B5, DR1, DR4, autoimmune disease, and Eales disease etiologies [6,12,14].

A diagnosis of Eales disease is based on a multimodal approach that includes a thorough examination of the patient’s history, clinical findings, and laboratory tests. Eales disease is considered to be a diagnosis of exclusion as there is no specific diagnostic test. A wide variety of retinal disorders are associated with inflammation and capillary ischemia; it is essential to rule out other causes of retinal vasculitis. Referrals to other specialties may be needed to exclude other possible systemic diseases [17].

A differential diagnosis should include branch retinal vein occlusion, sickle cell retinopathy, diabetic retinopathy, Coat’s disease, sarcoidosis, tuberculosis, and syphilis [18].

The disease is most common in young male adults and it is usually bilateral. Asymmetrical involvement of the eyes has been reported; the disease can unilaterally manifest in the early stages, but usually both eyes are involved as the disease progresses [1,11,17]. Nicolcescu et al. reported the case of a 48-year-old woman with unilateral Eales disease. The particularity of this case was that the disease remained unilateral for 9 years after the onset [12]. Ivanescu et al. described the case of a 40-year-old woman diagnosed with bilateral Eales disease whose condition rapidly worsened postpartum, with recurrent vitreous hemorrhages and a limited response to systemic treatment. In this case, the disease was controlled using intravitreal sustained-release dexamethasone implants (Ozurdex) [13].

A retrospective study conducted by Murrilo Lopez et al. revealed that the most frequent reason for a first ophthalmological exam in patients with Eales disease was a decrease in visual acuity due to vitreous hemorrhages in previously asymptomatic patients [17]. Similarly, our patient was referred to our clinic due to a vitreous hemorrhage that reduced visual acuity without any other prior visual symptoms.

Our patient presented a bilateral involvement with important retinal fundus alterations by the time he was first referred to our clinic. Typical pathologic retinal changes described in Eales disease include peripheral vasculitis, capillary nonperfusion and new-vessel formation at the level of the disc and elsewhere [1,10]. A fundus examination revealed similar findings in our patient along with a vitreal hemorrhage and thick epimacular membrane. Lopes et al. reported similar findings in a series of three Eales disease cases [11].

Recurrent vitreous hemorrhages may appear at the proliferation stage due to the progression of the ischemia and new-vessel proliferation. Our patient exhibited hematic cells in the vitreous body at presentation; another hemorrhage was recorded one month later before the initiation of oral steroid and immunosuppression therapy. Pars plana vitrectomy was not required in our case as the hemorrhage spontaneously subsided within a few weeks and remained clinically stable.

Spectral domain OCT is useful in the diagnosis and follow-up of macular changes, as well as in the identification of abnormalities in the vitreoretinal interphase that could arise during the inflammation phase. Rajukah et al. noted that sixteen (51.6%) of their analyzed cases presented macular changes identified using spectral domain OCT [19]. Important macular changes were also revealed using spectral domain OCT in our case. The patient presented thick epimacular membranes (ERMs) in both eyes as well as mild traction in the right eye. A similar case was reported by Sanchez Vicente et al., who described the presence of vitreomacular traction and epimacular membranes in a 38-year-old male patient later diagnosed with Eales disease [20]. Posterior vitreous detachment was noted in both eyes, an aspect that has previously been described in patients with Eales disease [14,21].

Similar findings have been presented in recent studies. Goel et al. reported that 58.2% of the Eales disease cases analyzed in their study had a macular involvement. The most common finding was macular edema, followed by ERM [22]. The involvement of the macula arises due to the occlusive nature of Eales disease and peripheral vasculitis [23].

Management is dictated by the stage of Eales disease [24]. The first line of treatment in the early inflammatory stage is systemic steroids. Usually, oral corticotherapy (prednisolone 1 mg/kg/day) is initiated, followed by a progressive reduction in the dose over the course of several months [1,14].

Azathioprine may be used as a steroid-sparing agent in the case of significant side effects due to systemic corticosteroids, or in patients who do not respond to corticotherapy [11,14]. Saxena et al. evaluated the efficacy of combined oral corticosteroids and a pulsed low-dose oral methotrexate therapy in Eales disease. They concluded that this was clinically effective with an adequate safety profile [24]. Intravitreal triamcinolone may be used in the presence of macular edema. Intravitreal anti-vascular endothelial growth factor (VEGF) may be required due to neovascularization [1,9,10].

The treatment of choice in more advanced cases when the disease is in the proliferative stage is laser photocoagulation [13,14]. The aim of the laser treatment is to induce the regression of neovascularization in the areas of retinal non-perfusion. In cases where optic disc neovascularization is present, a pan-retinal photocoagulation laser is required.

Biswal et al. conducted a retrospective study that analyzed the visual outcomes of 500 patients with Eales disease after 10 years of follow up. They concluded that patients who received oral corticotherapy in the acute stage and/or laser photocoagulation had a significantly improved visual outcome at the final visit compared with those who did not undergo corticotherapy or laser treatment [25].

Our patient was treated with a combination of systemic corticosteroids, anti-tuberculosis therapy and immunosuppressive therapy with oral azathioprine to control the disease and to prevent side effects from prolonged corticosteroid treatment. Pan-retinal photocoagulation was also performed because our patient was in the proliferative stage.

Pars plana vitrectomy is necessary for cases with persistent vitreous hemorrhages, with or without associated retinal detachment [17]. Studies have reported a good prognosis regarding vitrectomy in Eales disease, likely due to the posterior vitreous usually detaching early from the retinal surface [26]. Early pars plana vitrectomy led to an improved visual outcome in cases with recurrent vitreous hemorrhages [27]. A worse visual prognosis was observed in patients who developed tractional retinal detachment as well as combined tractional and rhegmatogenous retinal detachment [28,29]. Bansal et al. reported that vasculitic tractional retinal detachment tended to be associated with peripheral fibrovascular proliferations and retinal folds, making surgical intervention more challenging [30]. An improved visual outcome may be achieved by a more experienced vitreoretinal team due to the complexity of the surgical cases associated with proliferation [26,31].

Possible complications associated with Eales disease include neovascular glaucoma, tractional retinal detachment, and cataracts. Neovascularization can lead to recurrent vitreal hemorrhages due to the ischemic nature of vasculitis. In the literature, two case reports associated Eales disease with neovascular glaucoma. Alfayyadh et al. described the case of a 24-year-old male who was admitted to an emergency room due to neovascular glaucoma symptoms who was subsequently diagnosed with proliferative Eales disease [32]. Similarly, Bleidele et al. reported the case of a 26-year-old male patient with bilateral Eales disease who developed aggressive neovascular glaucoma; this led to optic atrophy and total blindness in the left eye despite intensive treatment [9].

Eales disease carries a good visual prognosis with an early diagnosis, proper treatment and regular follow-ups. Vision loss is most commonly caused by recurrent vitreous hemorrhages, which are usually transitory [11]. Severe vision loss is usually caused by retinal detachment.

The particularity of our case was the association between the hepatitis B virus and Borrelia infection, which required investigation and treatment prior to the initiation of immunosuppression therapy. To our knowledge, this association has not been reported elsewhere. Studies have revealed an increased risk of HBV reactivation during corticosteroid therapy; thus, it is advisable to remain alert and regularly follow up with patients [33]. Taking into account all the associated pathologies, a multidisciplinary approach was required in our case to safely initiate the corticosteroid treatment and the subsequent immunosuppressive therapy with azathioprine. The presence of polyadenopathies raised the suspicion of lymph-node tuberculosis; a lymph-node biopsy was required prior to the immunosuppression treatment alongside the prophylactic isoniazid.

## 4. Conclusions

Eales disease is an idiopathic inflammatory venous occlusive disease that is characterized by perivascular phlebitis, peripheral non-perfusion, and neovascularization. It remains a diagnosis of exclusion established on the basis of clinical and angiographic aspects, as there is no specific diagnostic test.

Consequently, a multidisciplinary approach to the management of these patients is advisable to exclude other possible causes and to prevent further complications, as seen in our presented case. Our complementary medical therapy, alongside laser photocoagulation, enabled us to obtain an individualized care plan. This resulted in an optimal outcome for our patient.

In conclusion, it is important to consider a multidisciplinary approach when treating associated complex pathologies and to be mindful of the possible complications that could arise as side effects of the required treatment.

## Figures and Tables

**Table 1:** Infectious-disease panel for laboratory results of patient.

Laboratory Test (Units)	Results	Reference Range
Herpes simplex virus 1 IgG	94.86	0.0–0.6
Cytomegalovirus IgG (AU/mL)	143.50	0.0–6
Borrelia IgG	Positive	Negative
Borrelia IgM	Positive	Negative
Cytomegalovirus DNA (UI/mL)	Undetectable	Undetectable
HSV1 DNA (UI/mL)	Undetectable	Undetectable
Hepatitis virus B DNA (UI/mL)	835	Undetectable
Toxoplasma Gondii IgG (UI/mL)	0.10	0.0–1.6
Toxoplasma Gondii IgM	0.05	0.0–0.5
Anti-HIV1/2 antibodies	Absent	Absent
RPR carbon	Negative	Negative
Syphilis test treponemic Ag	Negative	Negative

HSV: herpes simplex virus; RPR: rapid plasma reagin.

**Table 2:** Summary of previous case reports of Eales disease.

Study	Population	Treatment	Results
1. Bleidele et al. [9]	A 26-year-old male patient with bilateral Eales disease	Corticosteroids, azathioprine, mycophenolate mofetil, 12 anti-VEGF injections, and laser treatment.	Total blindness in the left eye and legal blindness in the right eye.
2. Călugăru et al. [10]	A 39-year-old male patient with bilateral Eales disease	Pars plana vitrectomy and endolaser treatment (LE), laser scatter photocoagulation (RE).	20/20 visual acuity in both eyes.
3. Lopes et al. [11]	A 27-year-old male patient with unilateral Eales disease A 48-year-old male patient with bilateral Eales disease A 58-year-old male patient with bilateral Eales disease	Topical and systemic corticotherapy, quadruple anti-tuberculosis treatment, and methotrexate. Oral corticotherapy, quadruple anti-tuberculosis treatment, oral methotrexate, 11 anti-VEGF injections, and laser photocoagulation. Oral corticotherapy, quadruple anti-tuberculosis treatment, 3 anti-VEGF injections, and laser photocoagulation.	Clinically stable; no signs of active vasculitis after 2 years. 10/10 visual acuity in both eyes with oral methotrexate (20 mg/week) associated with prednisolone (2.5 mg/day). Clinically stable; BCVA was 10/10 (RE) and 8/10 (LE) after 4 years with no need for treatment.
4. Nicolcescu et al. [12]	A 48-year-old female patient with unilateral Eales disease	Laser photocoagulation.	Clinically stable; VA was 20/20 (RE) with no need for treatment.
5. Ivanescu et al. [13]	A 40-year-old female patient with bilateral Eales disease	Oral corticotherapy, azathioprine, mycophenolate mofetil, intravitreal dexamethasone implants (Ozurdex^®^, Allergan; Bucharest; Romania) in RE and LE, and vitrectomy with silicone oil tamponade.	VA was 20/20 (RE); light perception (LE).

## Data Availability

The authors declare that the data in this research are available from corresponding authors upon reasonable request.
